# Detection of human enteric viral genes in a non-native winter crane fly, *Trichocera maculipennis* (Diptera) in the sewage treatment facilities at Antarctic stations

**DOI:** 10.1186/s13071-024-06555-4

**Published:** 2024-11-24

**Authors:** Sook-Young Lee, Ji Hee Kim, Seunghyun Kang, Kye Chung Park, Sung Mi Cho, Carla Ximena Salinas, Lorena Rebolledo, Hugo A. Benítez, Tamara Contador Mejías, Alvaro Soutullo, Eduardo Juri, Sanghee Kim

**Affiliations:** 1 Division of Life Sciences, Korea Polar Research Institute, Incheon, Republic of Korea; 2 The New Zealand Institute for Plant and Food Research Ltd., Auckland, New Zealand; 3 Departamento Científico, Instituto Antártico Chileno, Punta Arenas, Chile; 4 Millennium Institute Biodiversity of Antarctic and Subantartic Ecosystem (BASE), Santiago, Chile; 5 Cape Horn International Center (CHIC), Centro Universitario Cabo de Hornos, Universidad de Magallanes, Puerto Villiams, Chile; 6 Laboratorio de Ecología y Morfometría Evolutiva, Centro de Investigación de Estudios Avanzados del Maule, Universidad Católica del Maule, Talca, Chile; 7 Núcleo Milenio de Salmónidos Invasores (INVASAL), Concepción, Chile; 8 Centro Universitario Regional del Este, Universidad de la República, Montevideo, Uruguay; 9 Instituto Antártico Uruguayo, Montevideo, Uruguay

**Keywords:** *Trichocera maculipennis*, Non-native winter crane fly, Antarctica, Virus, Mechanical transmission

## Abstract

**Background:**

The Antarctic environment is susceptible to the introduction of non-native species due to its unique ecosystem, which has evolved under geographical isolation and extreme climatic conditions over an extended period. The recent introduction of the non-native winter crane fly, *Trichocera maculipennis*, to maritime Antarctica may pose a potential threat to the Antarctic ecosystem. In this study, we evaluated the possibility of the mechanical transmission of viruses by *T. maculipennis*.

**Methods:**

We assessed the potential for the mechanical transmission of viruses using next-generation sequencing (NGS), quantitative PCR (qPCR), and virus isolation methods from *T. maculipennis* (Tm)-related samples (Tm body-wash fluid and Tm body-ground samples) collected from habitats and sewage treatment facilities located at three research stations in Antarctica.

**Results:**

Virome analysis detected the genomic fragments of human adenovirus (AdV) and human endogenous retrovirus (HERV) in Tm-related samples. These viruses are commonly found in human feces. In addition, plant viruses, such as pepper mild mottle virus (PMMoV) and cucumber green mottle mosaic virus (CGMMV), both known indicators of enteric viruses, were identified in all Tm-related samples, likely originating from wastewater. However, the low quantities of AdV and HERV genomes detected in Tm-related samples through qPCR, coupled with the non-viability of AdV in virus isolation tests, indicate that *T. maculipennis* has limited potential for mechanical transmission under the conditions in the studies.

**Conclusions:**

Our study represents the first evaluation of the potential risk of non-native species serving as vectors for viral pathogens in Antarctica. Although the viruses detected were in relatively low quantities and non-viable, this study highlights the importance of further evaluating the risks associated with non-native species, particularly as the likelihood of their introduction increases to Antarctica due to climate change and increased human activity.

**Graphical abstract:**

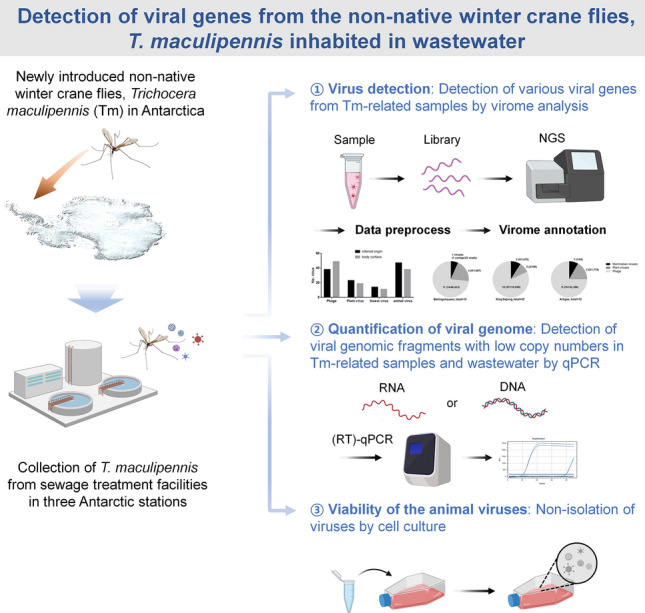

**Supplementary Information:**

The online version contains supplementary material available at 10.1186/s13071-024-06555-4.

## Background

Antarctica is less exposed to non-native species than other continents due to its geographical isolation and extreme climatic conditions. As a result, the Antarctic terrestrial ecosystem exhibits a notable scarcity of biodiversity owing to the survival of only a limited number of native species [1]. However, recent increases in human activity and climate change have led to the introduction of non-native species, some of which have managed to establish themselves within Antarctic communities [2]. The majority of non-native species introduced into Antarctica are primarily distributed in the sub-Antarctic region. Among these, the recently discovered *Trichocera maculipennis* (Diptera), a non-native fly (NNF), represents a significant example of a species that poses significant challenges to eradicate [1, 3]. This species originated in the Northern Hemisphere and was initially introduced to King George Island, one of the maritime Antarctic South Shetland Islands, in 2006 [4, 5]. Since then, it has been reported within or in the vicinity of several research stations, including the Uruguayan Artigas station in 2006 [4], Chilean Frei station in 2009/2010, Korean King Sejong station in 2013/2014 [6], Polish Arctowski station in 2017 [5, 7] and Russian Bellingshausen station in 2018/2019 [3, 8]. The populations found at each research station exhibited two different lineages, implying that they had at least two geographic origins and were introduced by multiple events [9]. The risk of further introductions of non-native species, including *T. mculipennis*, transmitting pathogens has not yet been fully assessed.

Arthropods, especially insects, are considered potential vectors of disease transmission due to their ability to fly, which facilitates mechanical transmission. Mechanical transmission involves the passive acquisition of pathogens by vectors, which can then carry them temporarily either internally or on their external surface, before spreading them to other hosts. Understanding the role of vectors in pathogen transmission is critical for assessing the potential risks to public health and ecosystem stability. Recent studies have identified a range of pathogens, including viruses, bacteria, fungi and parasites, on the external surfaces of insects, including influenza viruses, coronaviruses, *Campylobacter*, *Salmonella*, *Cladosporum*, *Aspergillus*, *Ascaris*, *Entamoeba*, and others [10]. Research has specifically focused on houseflies, assessing their potential as vectors of infectious diseases in livestock. Under laboratory conditions, the ability of houseflies to carry and transmit several subtypes of avian influenza viruses, including H5, H7 and H9, has been confirmed [11–13]. Furthermore, laboratory studies have shown that porcine reproductive and respiratory syndrome virus (PRRSV), Newcastle disease virus and Orf virus, which cause infectious diseases in livestock, may be mechanically transmitted through vectors [14–16]. In particular, severe acute respiratory syndrome coronavirus 2 (SARS-CoV-2), which is responsible for the 2019 global pandemic, has been found to remain infectious in houseflies for up to 24 h post-exposure [17, 18]. This potential for mechanical transmission is further supported by the field studies, which have detected various animal infectious disease viruses, including SARS-CoV-2, in livestock farms and on flies collected outdoors [19–22]. In this context, the aim of the present study was to evaluate the potential of the NNF *T. maculipennis,* recently introduced to Antarctica and inhabiting sewage treatment facilities at Antarctic research stations, to act as a mechanical vector of viral pathogens.

## Methods

### Sample preparation

**Fig. 1:**
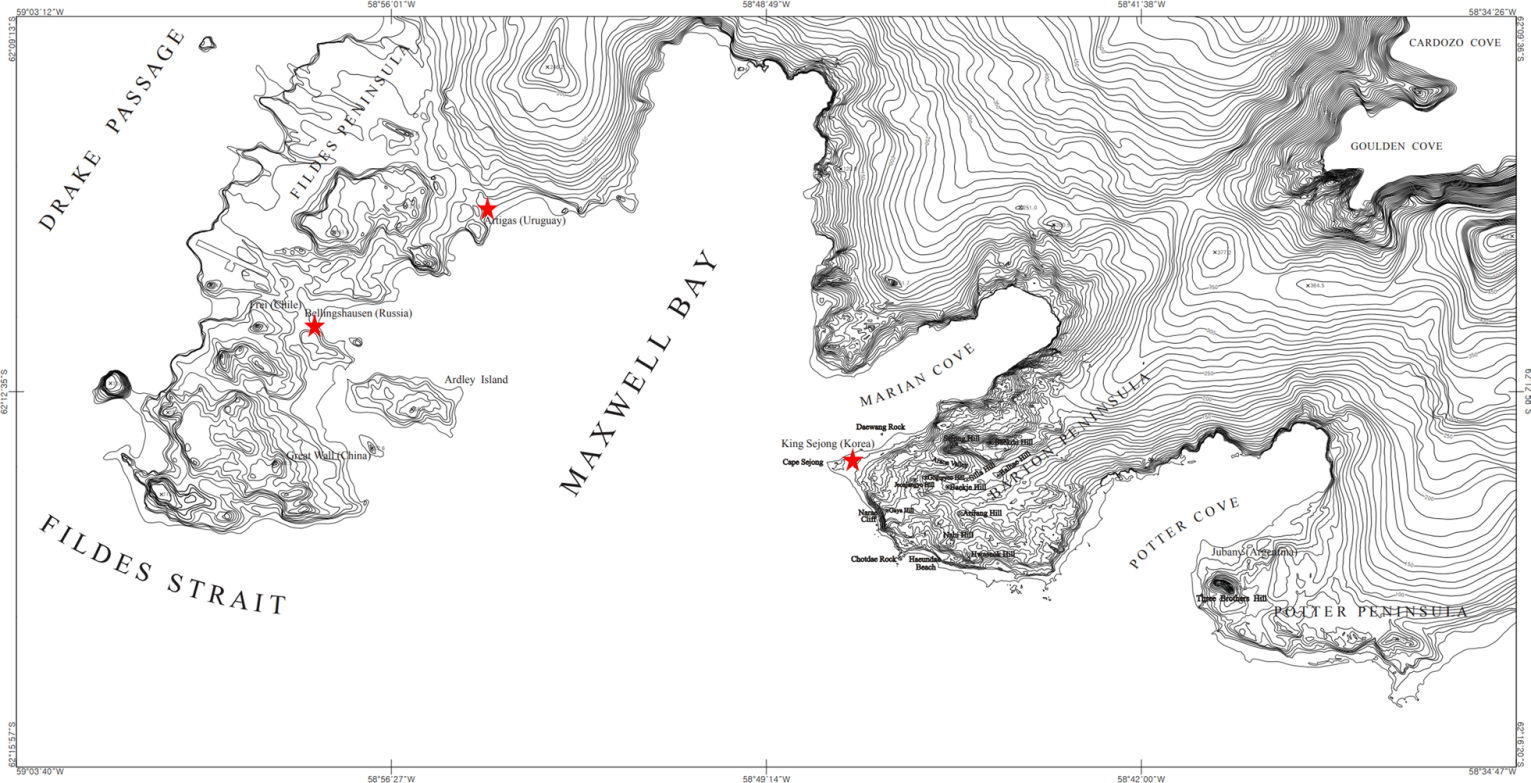
Collection sites of non-native winter crane flies (*Trichocera maculipennis*). The three research stations of Antarctica included in the study are marked with an asterisk. From left to right, the Bellingshausen station (Russia), Artigas station (Uruguay) and King Sejong station (Republic of Korea)

*Trichocera maculipennis* flies were collected using UV traps from sewage treatment facilities at three Antarctic research stations (Russian Bellingshausen station (62°11′55″S 58°57′38″W), Korean King Sejong station (62°13′22″S 58°47′18″W) and Uruguayan Artigas station (62°11′04″S 58°54′14″W)) located on King George Island, Antarctica (Fig. 1). UV traps were installed in the wastewater facilities at each station for 3–4 days. Ten flies were collected from each station and immediately washed in 3 ml of phosphate-buffered saline (PBS) containing 1% antibiotic–antimycotic solution (Corning Inc., Corning, NY, USA). The washing fluid samples from the body surface of each *T. maculipennis* (Tm body-wash fluid sample) were centrifuged at 3000 rpm for 5 min at 4 °C. Subsequently, the supernatant from each sample was filtered through a 0.45-µm membrane filter (Merck Millipore, Burlington, MA, USA) to remove bacteria and large particles. To validate the viral sequences identified in the *T. maculipennis* (Tm) body-wash fluid and Tm body-ground samples (hereafter, the two sample types are referred to in terms of Tm-related samples), we utilized 2 l samples of both influent and effluent wastewater collected from the sewage treatment facilities at King Sejong station. All samples were stored at − 20 °C and shipped to a laboratory in South Korea by sea. Upon arrival, the fly samples were homogenized by TissueLyser II (Qiagen, Hilden Germany), and the supernatant from ground flies (Tm body-ground sample) was then collected after centrifugation at 13,000 rpm for 5 min at 4 °C. Tm-related samples were processed for viral gene detection following the methodology outlined by Wu et al. [23]. Naked nucleic acids were digested with a cocktail of DNase and RNase enzymes, and viral nucleic acids were extracted using the QIAmp MinElute Virus Spin Kit (Qiagen).

### Next-generation sequencing

Complementary DNA (cDNA) and RNA libraries were synthesized from the samples using a QIAseq FX Single Cell RNA Library Kit (Qiagen) following the manufacturer’s instructions. To measure the concentrations and the length of the cDNA library, we used LightCycle qPCR (Roche, Basel, Switzerland) and the Agilent High Sensitivity D5000 ScreenTape system (Agilent Technologies, Inc., Santa Clara, CA, USA), respectively. The quantified libraries were then sequenced to 3 Gb using an Illumina HiseqX 150PE instrument (Illunina Inc., San Diego, CA, USA) according to the manufacturer’s recommendations. Sequencing reads were quality-trimmed (Q ≥ 30, length ≥ 50 bp) using Trim Galore (Babraham Bioinformatics, Babraham Institute, Cambridge, UK) and then classified with DeconSeq ver. 0.4.3 utilizing a database sourced from the National Center for Biotechnology Information (NCBI) with a coverage threshold of 70% and identity threshold of 90%. Contig assembly was performed using the SPAdes assembler ver. 3.14.1, followed by mapping with BWA-mem v0.7.17, and viral contig annotations were identified using BLASTn [24–26].

### Concentration of wastewater samples in King Sejong station

The wastewater samples stored at − 20°C for approximately 6 months were thawed overnight at 4 °C. We employed a modified polyethylene glycol (PEG) precipitation concentration method as previously described by Sapula et al. [27]. Briefly, 250 ml of influent and effluent water samples were centrifuged for 30 min at 5000 *g* to precipitate large particles. Subsequently, 200 ml of the supernatant was transferred to 250-ml PPCO centrifuge bottles (Thermo Fisher Scientific, Waltham, MA, USA) followed by the addition of 7% PEG6000 with 0.4 M NaCl. This mixture was gently agitated for 2 h at 4 °C. Subsequently, the solution was transferred to 50-ml PPCO centrifuge tubes (Thermo Fisher Scientific) and centrifuged for 90 min at 12,000 *g* and 4 ° to precipitate the pellet, following which the supernatant was discarded and the pellet was resuspended in 1 ml PBS. Finally, viral nucleic acids were extracted using an AllPrep PowerViral DNA/RNA kit (Qiagen).

### Quantification of viral genomes

To quantify mammalian viral genes, we designed primers and probes based on the sequence of human adenoviral and human endogenous retroviral contigs (see Additional file 1: Table S1). For absolute quantitation by real-time (RT)-qPCR, a standard curve was plotted using plasmids containing 123-bp and 218-bp fragments of the adenovirus (AdV) and human endogenous retrovirus (HERV) genomes, respectively. The concentration of each viral genome was measured using a Nanodrop spectrophotometer (Thermo Fisher Scientific), and the gene copy number in the standard was calculated using the following formula: (DNA amount [ng] × 6.0221 × 10^23^ molecules/mole)/ ((DNA length × 660 g/mole) × 1 × 10^9^ ng/g). A standard curve was generated using a decimal serial dilution of the plasmid, including each viral gene, and the threshold cycle values (Ct values) were plotted against the log concentrations of the copy numbers. Calibration curves for AdV (*y* = − 0.248x + 11.55,* R*^2^ = 0.996) and HERV (*y* = − 0.245*x* + 13.24,* R*^2^ = 0.999) revealed a linear dynamic range between 4.51 × 10^3^ and 4.51 × 10^8^.

The copy number of each viral genome was measured from 5 μl of total nucleic acid extracted in the Tm body-wash fluid and Tm body-ground samples using the commercial reagents TOPreal One-step qPCR/RT-qPCR Kit (Enzynomics, Daejeon, Korea) on the CFX Real-Time System (Bio-Rad Laboratories, Hercules, CA, USA). For AdV, the PCR conditions were: 95 °C for 10 min, 45 cycles at 95 °C for 5 s and 60 °C for 30 s. For HERV, the PCR conditions were: 1 cycle at 50 °C for 30 min, 1 cycle at 95 °C for 10 min and 45 cycles at 95 °C for 5 s and 60 °C for 30 s. Copy numbers of the samples were calculated using the standard curve method.

### Propagation of viruses

To determine the propagation of the detected viruses, we inoculated 200 µl of both Tm body-wash fluid and Tm body-ground samples into monolayer-cultured A549 cells (human lung carcinoma [CCL-185] line). The cells were cultured in Dulbecco’s modified Eagle’s medium (DMEM) supplemented with 5% fetal bovine serum (FBS) and 100 U/ml penicillin–streptomycin. The culture was then incubated at 37 °C in a humidified atmosphere containing 5% CO_2_ [28, 29]_._ After 5 to 7 days, the propagation of the detected viruses in the inoculated cells was confirmed by quantifying each viral gene using the methods described in the section Quantification of viral genomes.

## Results

### Next-generation sequencing data analysis

Six metagenomic datasets were obtained from the Tm-related samples (Tm body-wash fluid and Tm body-ground samples) collected from the sewage treatment facilities at the Russian Bellingshausen, Korean King Sejong and Uruguayan Artigas stations. The raw datasets were processed by trimming sequences with a base quality of ≥ 30 and a minimum length of 50 bp. Trimmed data ranging from 30,132,374 to 31,650,580 sequences were analyzed for viral sequences. From these, 3966 to 143,369 viral reads were extracted based on coverage of ≥ 70% and identities of ≥ 90% using the NCBI virus database as a cut-off score. In each dataset, at least 16 contigs of ≥ 200 bp and one to three contigs of ≥ 1000 bp were identified. Subsequent BLAST analysis using the NCBI VirDB confirmed the presence of between 17 and 53 viral contigs (Rank1, e-value ≤ 1e^−5^), as detailed in Additional file 1: Table 2S.

### Detection of various viruses by next-generation sequencing

**Fig. 2:**
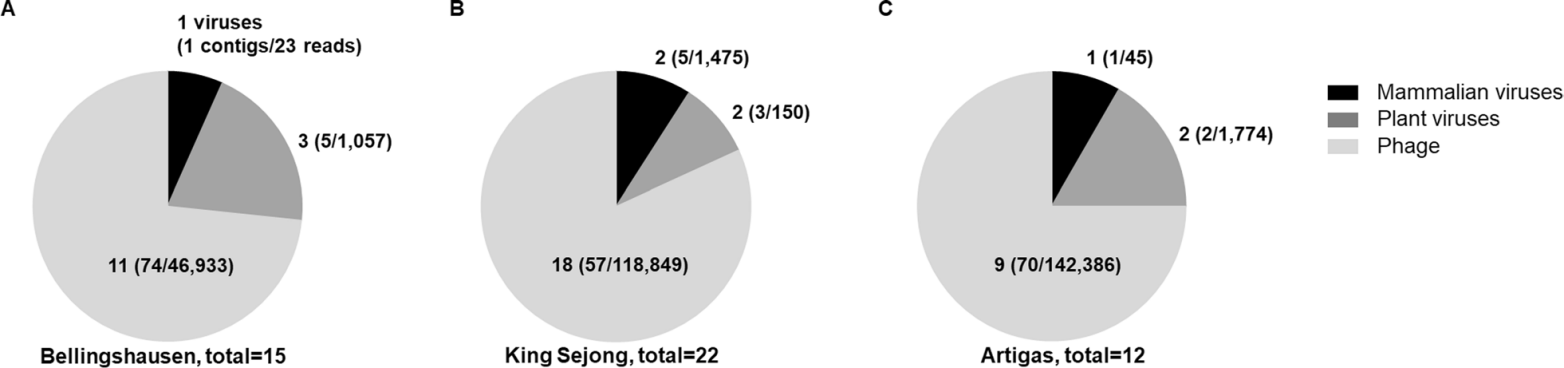
Number of viruses, viral contigs and viral reads detected in Tm-related samples collected from each Antarctic station. **A** Bellingshausen station (Russia), **B** King Sejong station (Republic of Korea) and **C** Artigas station (Uruguay). Tm-related samples, *Trichocera maculipennis* body-wash fluid and *T. maculipennis* body-ground samples

One mammalian and three plant viral genomic fragments were detected in the Tm-related samples (Tm body-ground and Tm body-wash fluid samples) collected from the Bellingshausen station. At the King Sejong station, one mammalian and one plant viral sequences were found in the Tm body-ground and Tm body-wash fluid samples, respectively. A total of two mammalian and two plant virus sequences were found. In the Artigas station samples, one mammalian and two plant viral sequences were also detected (Fig. 2; Additional file 1: Table S3). Among the viral sequences found in the Tm-related samples were mammalian viruses, such as AdV and HERV, as well as plant viruses, such as the pepper mild mottle virus (PMMoV) and cucumber green mottle mosaic virus (CGMMV) (Additional file 1: Table S4).

### Detection of mammalian viruses

Two mammalian viruses, AdV and HERV, were identified in Tm-related samples collected from the sewage treatment facilities at the three Antarctic stations. AdV geomic fragments were found in a Tm body-ground sample from Bellingshausen station and a Tm body-wash fluid sample from King Sejong station. Additionally, HERV gene fragments were detected in a Tm body-ground sample from King Sejong station and a Tm body-wash fluid sample from Artigas station. In the Tm body-ground sample from Bellingshausen station, a 235-bp contig showed approximately 80% coverage and 100% sequence identity with the *E1B* 55-kDa protein gene of human AdV-1 (Accession no. AC_000017). Similarly, a 381-bp adenoviral contig in the Tm body-wash fluid sample collected from King Sejong station exhibited 99.7% sequence identity with the *E1B* 55-kDa protein gene of human AdV-C (Accession no. NC_001405). Partial HERV sequences were also identified in the Tm body-ground sample collected from King Sejong station, showing 97.9% coverage (1362/1347 bp) and 92.7–95.7% sequence identity with the *Pol* and *Env* genes of HERV K113 (Accession no. NC_022518). Furthermore, a 434-bp HERV sequence in the Tm body-wash fluid sample from the Artigas station was 95.7% identical to the *Gag* gene sequence of HREV K113 (Accession no. NC_022518), as detailed in Table 1 and Additional file 1: Table 4S.

**Table 1:** Contig annotation of mammalian viruses detected from each sample

Site/station	Sample	Virus	Reference accession no.	Target gene	Coverage (%)	Length/matches (bp)	Identity (%)
Bellingshausen	Tm body-ground^a^	Human AdV-1	AC_000017	*E1B-55 K*	80.4	235/189	100
King Sejong	Tm body-ground	HERV	NC_022518	*Pol*,* Env*, and LTRs	97.9	1362/1347	92.7–95.7
	Tm body-wash^b^	Human AdV-C	NC_001405	*E1B-55 K*	99.7	381/336	99.7
Artigas	Tm body-wash	HERV	NC_022518	*Gag*	95.9	434/416	95.7

*AdV* Adenovirus,* HERV* human endogenous retrovirus,* LTR* long terminal region,* Tm* *Trichocera maculipennis*

^a^*T. maculipennis* body-ground sample

^b^*T. maculipennis* body-wash fluid sample

### Quantification of viral genome in Tm-related and wastewater samples

To quantify viral genomes in the Tm-related samples, we designed primer/probe sets targeting the *E1B* and *Pol* genes of AdV and HERV based on their respective contig sequences. In the Tm body-wash fluid sample from King Sejong station, the adenoviral genome was detected at a concentration of 1.35e + 2.36 copies/µl. However, it was not detected in the Tm body-ground sample from the Bellingshausen station. Similarly, the HERV genome was not found in the Tm body-ground and Tm body-wash fluid samples from the King Sejong and Artigas stations. In the concentrated influent and effluent samples from King Sejong station, the AdV genome was also detected, with viral copy numbers of 1.35e + 2.16 copies/µl and 1.35e + 2.31 copies/µl, respectively (Table 2).

**Table 2:** Adenoviral and retroviral quantification from each sample

Site/station	Sample	Real-time qPCR		Virus propagation
		AdV	HERV	
Bellingshausen	Tm body-ground^a^	–^c^	–	NT
King Sejong	Tm body-ground	–	–	–
	Tm body-wash^b^	1.35e + 2.36 copies/μl	–	–
	Influent	1.35e + 2.16 copies/μl	–	NT
	Effluent	1.35e + 2.31 copies/μl	–	NT
Artigas	Tm body-wash	–	–	NT

*AdV* Adenovirus,* HERV* human endogenous retrovirus, *NT* Not tested,* Tm Trichocera maculipennis*

^a^*T. maculipennis* body-ground

^b^*T. maculipennis* body-wash fluid

^c^–, Not detected

### The infectious potential of viruses detected in Tm-related samples and wastewater

The inoculated cells were incubated for 5 days, during which their physical characteristics were monitored daily. However, no significant changes were observed compared to control cells (normal cells) in their shape or adhesion properties (Additional file 2: Figure S1). Additionally, no viral genomes were detected in either the inoculated cell lysate or supernatant.

## Discussion

Insects are often considered to be primary or intermediate hosts of human disease agents because of their ability to mechanically transmit diseases by harboring pathogens on their external surfaces and within their internal organs [5, 7].* Trichocera maculipennis*, which originated in the Northern Hemisphere, was recently discovered in Antarctica, introduced to Antarctic stations 20 years ago [4, 5, 9]. As a prominent non-native species, its eradication in Antarctica presents challenges [6, 8]. Given the uncertain impact of non-native species on the Antarctic ecosystem, this study aimed to assess the potential role of this NNF as a vector for viral transmission.

The virome analysis of Tm-related samples (Tm body-wash fluid and Tm body-ground samples) revealed the presence of several plant and mammalian viral genomic fragments. Plant viral genomes, such as PMMoV and CGMMV, were detected along with the mammalian viruses AdV and HERV. PMMoV and CGMMV, which were found in all Tm-related samples, infect plants and cause crop diseases [30]. These plant viruses, which are commonly detected in human feces and wastewater, serve as indicators of enteric viruses and for microbial source tracking in wastewater treatment [31–34]. As these plant viruses possess exceptional stability in water and diverse environmental conditions, their presence in Tm-related samples is thought to originate from wastewater, suggesting that *T. maculipennis* may harbor diverse sewage pathogens.

AdV and HERV, detected in both Tm body-wash fluid and Tm body-ground samples from the three Antarctic research stations, are important mammalian viruses. AdV, which is associated with gastroenteritis in young children, is often transmitted via the oral-fecal route and is frequently found in wastewater [35–37]. HERV, an endogenous retrovirus inserted into the human germ cell DNA, is commonly found in the human gut and feces [38–40]. Although no other animal RNA viruses were detected, the genomes of AdV and HERV, which are relatively more resistant to degradation than RNA viruses, seem to be more easily detected in *T. maculipennis* wash fluid or lysates because they can remain preserved in environmental samples for a prolonged period [41].

A quantification test for adenoviral and retroviral genes (*E1B* gene of AdV and *Pol* gene of HERV) to assess their abundance in Tm-related samples indicated a very low quantity of viral genomes: 1.35e + 2.36 copies/µl in Tm body-wash fluid sample from the King Sejong station. Although the concentrations of these viral genes were near the detection limits, we concluded that *T. maculipennis* carried these viral genomic fragments at very low concentrations. This conclusion is based on the consistently negative results in non-template controls and the fact that our analysis spanned a dynamic range of concentrations up to e + 3 in the standard curves (Additional file 3: Figure S2) [42]. The presence of only a small amount of AdV in wastewater samples (1.35e + 2.16 copies/µl [influent] and 1.35e + 2.31 copies/µl [effluent]) suggested that the viral genes in *T. maculipennis* originated from the habitat at King Sejong station. However, the AdV gene was not detected in Tm body-ground samples from the Bellingshausen station, and HERV was absent in Tm body-ground or Tm body-wash fluid samples from the King Sejong and Artigas stations. Previous laboratory studies have shown that the concentration of infectious viruses and the duration of their external exposure are crucial factors in enabling mechanical transmission by vectors [12, 13]. For example, research on the mechanical transmission of influenza virus by houseflies indicates that low viral concentrations, high incubation temperatures and prolonged incubation reduce the persistence of influenza viruses [11–13]. In our study, while the duration of viral exposure on *T. maculipennis* was not precisely measured, the minimal amount of viral genomes and extremely low virus viability observed in the samples indicate limited transmission potential. However, given the number of flies used and the uncertain duration of environmental exposure, it is not possible to definitely rule out the possibility that *T. maculipennis* could serve as a vector under conditions more conductive to mechanical transmission.

The limited detection of AdV and the absence of HERV in the concentrated wastewater samples may be attributed to the long duration of transportation from Antarctica to the laboratory, despite storage at − 20 °C, or the presence of only viral genomic fragments, i.e. absence of infectious virus, in sewage treatment facilities at Antarctic research stations. Notably, long-term storage and repeated freezing and thawing of liquid wastewater can reduce the measurable concentrations of viral nucleic acids, especially RNA [43, 44]. Recent studies suggest that storing wastewater solids, like RNA extract or concentrate, at − 20 °C for approximately 4 months yields more reliable viral RNA quantification compared to storing unprocessed raw wastewater at 4 °Cor − 80 °C [45, 46]. Therefore, for more accurate quantification of viruses in Antarctic wastewater in the future studies, it would be essential to pre-process or concentrate wastewater prior to shipment from Antarctica. Another limitation of this study is the potential degradation of viral particles or infectious viruses within the wastewater treatment system, which may have resulted in the limited detection of viral genes in this study.

Although our study detected only modest amounts of the virus in Tm-related and wastewater samples, these findings should be interpreted with caution, particularly given the low viral concentrations and the possibility that the viruses detected were not viable. Nevertheless, this study represents the first assessment of the potential risk posed by NNFs (*T. maculipennis*) introduced into the Antarctic as a potential vector for pathogens. It remains critical to monitor non-native species in Antarctica as human activity and climate changes increase the likelihood of new introductions. Our study contributes to the establishment of risk assessment methods for non-native Antarctic species and offers a framework for diverse evaluations in future research.

## Conclusions

This study explored the potential of the NNF *T. maculipennis*, found in the sewage treatment facilities of three Antarctic research stations, to mechanically transmit viruses. A key finding was the detection of various viral genome fragments in *T. maculipennis*, including those of the human adenovirus and human endogenous retrovirus, which are commonly found in human feces and wastewater. However, the amounts of viral particles were minimal and the viruses non-viable. Nevertheless, due to the limitations of this study, particularly the low viral concentrations, it is not possible to definitely rule out the potential for *T. maculipennis* to act as a vector under conditions more conducive to mechanical transmission. This is the first study to assess the risk posed by *T. maculipennis* as a vector of pathogens in Antarctica. The results contribute to a broader evaluation of the risk of mechanical transmission of viruses by NNFs, providing insight into the potential implications of their future introduction.

## Supplementary Information

**Additional file 1: Table S1.** Primer information for quantification of viral genes (AdV and HERV). **Table S2.** The results of NGS data analysis from each data set. **Table S3.** Number of viral contigs detected from each site. **Table S4.** Information of the sequences of mammalian and plant virus obtained through NGS.

**Additional file 2: Supplementary Figure 1.** Cell culture for the viral propagation test using PBS and body-wash fluid sample. **A** A549 (human lung carcinoma, CCL-185) cell inoculated with 200 µL of PBS buffer (5 days after inoculation), and **B** A549 cell inoculated with 200 µL of Tm body-wash fluid of *T. maculipennis* collected at King Sejong station (5 days after inoculation).

**Additional file 3: Supplementary Figure 2.** Standard curves for the quantification of adenoviral and retroviral genome. **A** Adenoviral standard curve using plasmid containing adenoviral contig sequence, and **B** Retroviral standard curve using plasmid containing retroviral contig sequence.

## Data Availability

Illumina HiseqX 150PE data have been deposited in the GenBank Sequence Reads Archive (SRA) under the accession number PRJNA1052610.

## Abbreviations

AdV: Adenovirus
CGMMV: Cucumber green mottle mosaic virus
DMEM: Dulbecco’s modified Eagle’s medium
FBS: Fetal bovine serum
HERV: Human endogenous retrovirus
NNF: Non-native fly
PEG: Polyethylene glycol
PMMoV: Pepper mild mottle virus
Tm-related sample: *Trichocera mculipennis* body wash fluid and *T. maculipennis* body ground sample
qPCR: quantitative PCR
RT- PCR: quantitation by real-time
