# Supplementary figures and images for: Detection of human enteric viral genes in a non-native winter crane fly, Trichocera maculipennis (Diptera) in the sewage treatment facilities at Antarctic stations
Source: Parasit Vectors. 2024 Nov 24;17:485. doi: 10.1186/s13071-024-06555-4 (PMC11587659; doi:10.1186/s13071-024-06555-4)

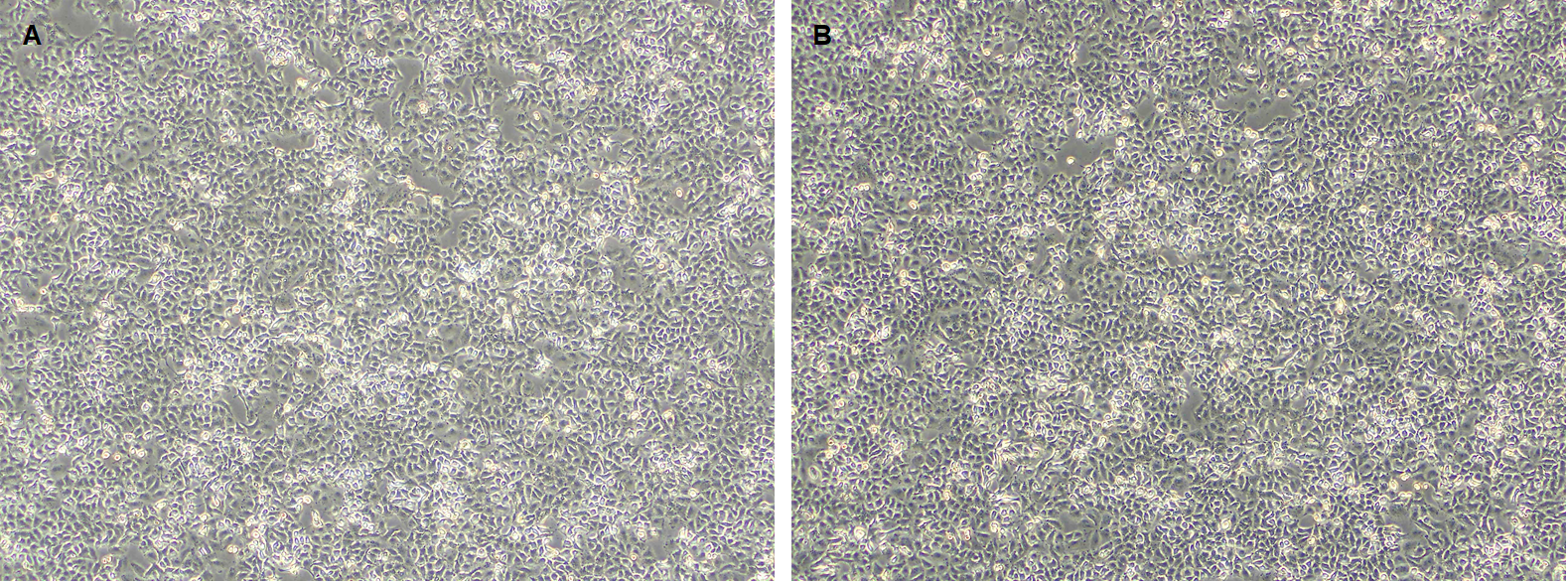

Supplement: Supplementary file 2 — Additional file 2: Supplementary Figure 1. Cell culture for the viral propagation test using PBS and body-wash fluid sample. A A549 (human lung carcinoma, CCL-185) cell inoculated with 200 µL of PBS buffer (5 days after inoculation), and B A549 cell inoculated with 200 µL of Tm body-wash fluid of T. maculipennis collected at King Sejong station (5 days after inoculation). [file 13071_2024_6555_MOESM2_ESM.tif]

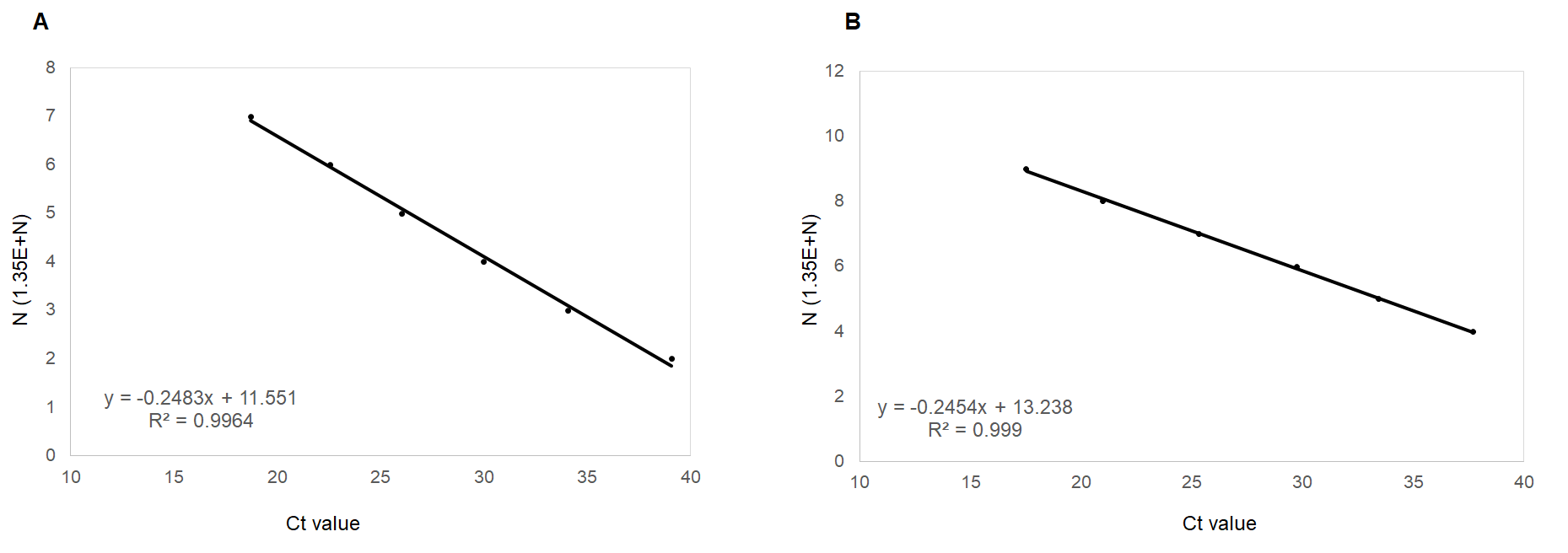

Supplement: Supplementary file 3 — Additional file 3: Supplementary Figure 2. Standard curves for the quantification of adenoviral and retroviral genome. A Adenoviral standard curve using plasmid containing adenoviral contig sequence, and B Retroviral standard curve using plasmid containing retroviral contig sequence. [file 13071_2024_6555_MOESM3_ESM.tif]
